# Prognostication of Primary Tumor Location in Early-Stage Nodal Diffuse Large B-Cell Lymphoma: An Analysis of the SEER Database

**DOI:** 10.3390/cancers13163954

**Published:** 2021-08-05

**Authors:** Yi Xia, Silan Huang, Yu Wang, Dexin Lei, Yanlou Wang, Hang Yang, Yan Gao, Panpan Liu

**Affiliations:** 1 Department of Medical Oncology, Sun Yat-sen University Cancer Center, 651 Dong Feng East Road, Guangzhou 510060, China; xiayi@sysucc.org.cn (Y.X.); huangsl@sysucc.org.cn (S.H.); wangyu@sysucc.org.cn (Y.W.); leidx@sysucc.org.cn (D.L.); wangyl4@sysucc.org.cn (Y.W.); yanghang@sysucc.org.cn (H.Y.); 2 State Key Laboratory of Oncology in South China, Guangzhou 510060, China; 3 Collaborative Innovation Center for Cancer Medicine, Guangzhou 510060, China

**Keywords:** early-stage nodal diffuse large B cell lymphoma, SEER database, survival, primary site, enrichment analysis

## Abstract

**Simple Summary:**

Few studies have examined the impact of primary tumor location on clinical outcome in patients with early-stage nodal diffuse large B-cell lymphoma (DLBCL). The objective of this study was to identify the association between primary tumor location and early-stage nodal DLBCL patient prognosis using a large population-based cohort and make an effort to uncover its underlying molecular mechanism using a public database. Our result shows that the prognosis of early-stage nodal DLBCL patients with tumors growing under the diaphragm is poorer. After screening DEGs and carrying out enrichment analysis, we found early-stage nodal diffuse large B-cell lymphoma located in different sites having different genetic characteristics. These results emphasize the importance of the primary tumor site on clinical decision-making and prognosis of patients with early-stage nodal diffuse large B-cell lymphoma.

**Abstract:**

The prognostic role of primary tumor location for clinical outcomes of patients with early-stage nodal diffuse large B-cell lymphoma (DLBCL) remains uncertain. We evaluated the relationship between primary tumor site and overall survival (OS) in 9738 early-stage nodal DLBCL patients from the Surveillance, Epidemiology, and End Results (SEER) database. The primary site of the tumors was characterized as supradiaphragm and subdiaphragm according to the definition of lymph node distribution in the Ann Arbor staging. The OS was significantly better for patients of the supradiaphragm group (*n* = 6038) compared to the ones from the subdiaphragm group (*n* = 3655) (hazard ratio (HR) 1.24; 95%CI: 1.16–1.33; *P* < 0.001), and it was preserved after propensity score matching (PSM) (HR 1.15; 95% CI: 1.07–1.24; *P* < 0.001). Gene enrichment analyses demonstrated that the subdiaphragm group has an upregulated extracellular matrix (ECM)-related signaling, which reportedly can promote growth, invasion, and metastasis of the cancer, and downregulated interferon response, which is considered to have anti-tumor function. Our results indicate the two tumor locations (supradiaphragm and subdiaphragm) presented different prognostic implications for the overall survival, suggesting that the tumor’s location could serve as a prognostic biomarker for early-stage nodal DLBCL patients.

## 1. Introduction

The diffuse large B-cell lymphoma (DLBCL) is the most common subtype of non-Hodgkin’s lymphoma (NHL), accounting for approximately 25 percent of NHL cases [1]. It is highly heterogeneous in terms of clinical presentation, morphology, genetics, and biologic behavior. About 25% to 40% of the DLBCL patients are observed early-stage (Ann Arbor stage I and II) disease [2,3]. The most widespread therapy for early-stage DLBCL is chemoimmunotherapy with R-CHOP (rituximab, cyclophosphamide, doxorubicin, vincristine, and prednisone) combined with or without radiation therapy (RT), which has greatly improved the outcome of DLBCL [4,5,6]. The ultimate goal for treatments of the early-stage DLBCL is cure of the disease or long-term survival. Despite the fact that early-stage DLBCL patients generally present favorable prognosis, about 30% of them is expected to relapse [7]. It is vital to explore the factors that can predict recurrence and chemotherapy resistance, and to identify the factors that can affect the prognosis of early-stage nodal DLBCL patients, aiming to contribute to disease progression prediction and support clinical decisions.

Significant progress has been recently achieved in understanding the biology of DLBCL and main advancements include the assessment of molecular risk through the evaluation of the cell of origin (COO) and the incorporation of specific genetic mutations into prognostication and clinical decision making. Recently, two whole-exome sequencing studies have suggested partially overlapping classification systems. Roland Schmitz et al. subdivided DLBCL into four subtypes on the basis of COO: MCD (based on the co-occurrence of MYD88^L265P^ and CD79B mutations), BN2 (based on BCL6 fusions and NOTCH2 mutations), N1 (based on NOTCH1 mutations), and EZB (based on EZH2 mutations and BCL2 translocations). Among them, BN2 and EZB subtypes have a favorable outcome and MCD and N1 subtypes have inferior outcomes [8]. Bjoern Chapuy et al. identified five groups defined as C1–C5, among which C1 and C4 harbor a favorable prognosis, while C3 and C5 have the worst outcome. These subtypes were described as follows: C1 exhibited BCL6 SVs in combination with mutations in NOTCH2 and immune escape and is similar to BN2 subtype; C2 is related to the chromosomal stability and cell cycle; C3, similar to the EZB subtype, harbored the same mutations in BCL2, EZH2, KMT2D, and CREBBP; C4 is characterized by mutations in SGK1, HIST1H1E, NFKBIE, BRAF, and CD83; C5 presented frequent mutations in CD79B and MYD88, which is similar to the MCD subtype [9]. Another five molecular subtypes were resolved by Stuart E. Lacy et al., annotated as MYD88, BCL2, SOCS1/SGK1, TET2/SGK1, and NOTCH2, along with an unclassified group. BCL2, NOTCH2, and MYD88 showed good, intermediate, and poor prognosis, respectively [10]. These molecular classifications have not only shown different gene expression characteristics, but also presented different responses to immunochemotherapy. Moreover, the coordinate genetic signatures were found to predict an outcome independent of the clinical international prognostic index (IPI), and suggest new combination treatment strategies. However, technical and financial difficulties limit the clinical application of the latter molecular classification.

Currently, the prognostic tools widely used in DLBCL are limited to the international prognostic index (IPI) with the variants used in it being the most commonly used ones. The IPI uses information regarding patient age, performance status, serum lactate dehydrogenase (LDH) level, disease stage, and degree of extranodal involvement to determine a score that correlates with the progression-free and the overall survival after standard therapy [11]. It is noteworthy that the IPI score system only includes the number of extranodal organs involved and does not consider the primary location of nodal DLBCL. Even in the enhanced IPI system, it only emphasized the prognostic importance of specific extranodal sites such bone marrow, lungs, brains, among others [12]. Except for the 25%~40% of DLBCL that are extranodal cases, most of the rest are nodal lesions [13,14,15,16]. Although some studies have demonstrated that the involvement of the extranodal tissue may lead to a worse prognosis for patients with nodal lesions [17,18,19], the prognosis of nodal patients in some parts, such as lymphomas originating from the abdomen, pelvis, or thorax [20], is even worse. A study based on the SEER database and the national cancer database (NCDB) was performed in an attempt to evaluate the relationship between lymph node lesions and prognosis in patients with early-stage DLBCL. Results showed that there were significant statistical differences in the survival rates among patients with DLBCL in different sites, and lymphomas originating from the abdomen, pelvis, or thorax showed higher risk genetic characteristics. It is suggested that the location of lymphadenopathy be included in the new prognosis score system of the early-stage DLBCL [20]. From this, we can find that there is indeed a difference in the prognosis of patients with early-stage DLBCL in the different sites, nodal or extranodal patients, and the influence of the primary site should be taken into consideration when making clinical decisions. The abovementioned authors divided the data into several groups and found that there was a statistical difference in the survival rate of patients with nodal DLBCL. However, there was no difference among groups, such as between abdomen and pelvic lymph nodes, thorax lymph nodes, and Waldeyer’s ring (WR), in prognosis. This kind of grouping may not be suitable for inclusion in the prognosis scoring system, and it is thus vital to know how to group the nodal DLBCL in different sites in order to obtain the best grouping mode.

Therefore, the focus of this study is to explore a feasible and simple grouping method for patients with early-stage nodal DLBCL based on the SEER database, that is, patients with nodal DLBCL in different primary sites are divided into the subdiaphragm group (Sub-DLBCL) and supradiaphragm group (Sup-DLBCL) according to the definition of lymph node distribution in Ann Arbor staging (still applicable to Lugano staging). We aim to explore the overall survival of patients with early-stage nodal DLBCL located on both sides of the diaphragm during the rituximab era, and try to identify the potential molecular mechanism between them, in an attempt to assist clinicians to better predict the prognosis of DLBCL patients with different parts and to provide a basis for clinical decision-making.

## 2. Materials and Methods

### 2.1. Patient Enrollment

**Figure 1:**
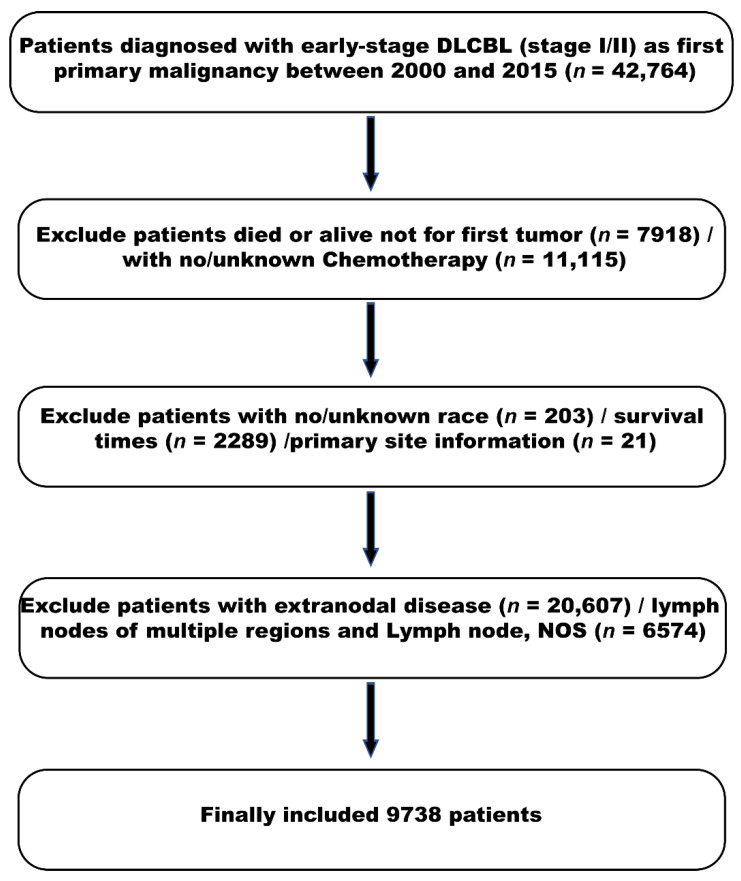
Flow chart of patient selection from the Surveillance, Epidemiology, and End Results (SEER) database.

In our cohort, the cases came from the registries of the SEER-9 cancer incidence file of the US National Cancer Institute from 1973 to 2015. We extracted cases of DLBCL from 2000 to 2014 using the ICD-O-3 code 9680, 9684/3 and 9688/3. Because our goal was to study the overall survival rate of early-stage nodal DLBCL patients on both sides of the diaphragm during the rituximab era, which was firstly approved in 1997, we only included patients diagnosed after 2000, which is a period that is expected to reflect widespread and quick use of rituximab in the treatment of lymphomas. We only included the nodal DLBCL patients and excluded patients with lymph nodes of multiple regions and lymph node, NOS. Only the stage I and stage II cases according to Ann Arbor staging with a defined primary site were included in our study. Patients that had died from reasons other than the primary tumor and patients with no survival time and no clear primary site were excluded. The detailed criterion for the inclusion and exclusion of patients is shown in Figure 1.

### 2.2. End Points

The primary endpoint of this study was the overall survival (OS). The OS was calculated from the beginning of the initial treatment until the time of death from any cause, or until the last follow-up time point.

### 2.3. Standard of Grouping

The primary site of nodal DLBCL is divided into the subdiaphragm (Sub-DLBCL) and supradiaphragm groups (Sup-DLBCL) according to the definition of lymph node distribution in Ann Arbor staging. The supradiaphragm group (Sup-DLBCL) includes lymph nodes of head, face, and neck/intrathoracic lymph nodes/lymph nodes of axilla or arm. The subdiaphragm group (Sub-DLBCL) includes intra-abdominal lymph nodes/pelvic lymph nodes/lymph nodes of inguinal region or leg. The Waldeyer’s ring and tonsil are the lymphoid tissue above the diaphragm and the spleen is the lymphoid tissue below the diaphragm.

### 2.4. Statistical Analysis

The baseline characteristics of the patients were compared among the different groups using Student’s t-test or an equivalent nonparametric test. The log-rank test was applied to compare the overall survival between different groups. The univariate Cox proportional hazard model was used to calculate hazard ratios (HR), stratifying for anatomical sites and adjusting for the following covariates: sex, age at diagnosis, race, and Ann Arbor stage. The 1:1 propensity score matching (PSM) was used to match the covariate proportions, mentioned above as covariates, with the cliper value being set to 0.05. Multivariate Cox regression analysis was used to explore the effect of matched primary site on its overall survival.

Statistical analysis was conducted using R programming environment version 3.6.2 (http://cran.r-project.org, accessed date: 20 March 2020), IBM SPSS Statistics version 25 (IBM Corp., Armonk, NY, USA), and GraphPad Prism version 8. *P*-values were all two-sided and statistical significance threshold was set at *P* < 0.05 if not mentioned. All confidence intervals were stated at the 95% confidence level.

### 2.5. Microarray Data

The gene expression profiles (RNA-sequencing expression levels) and clinical data were downloaded from The Cancer Genome Atlas (TCGA) database (https://cancergenome.nih.gov/, accessed date: 10 January 2021). Patients and samples with missing staging and primary location of the tumor were excluded. Ten supradiaphragm and four subdiaphragm patients were included from our study by applying the abovementioned exclusion criteria. This study was conducted in accordance with the publication guidelines of TCGA (http://cancergenome.nih.gov/publications/public action guidelines, accessed date: 10 January 2021).

### 2.6. Data Preprocessing and Differential Expression Genes Screening

R statistical software (version 3.6.2; https://www.r-project.org/, accessed date: 20 March 2020) and Bioconductor analysis tools (http://www.bioconductor.org/, accessed date: 20 March 2020) were utilized to process the raw data. The edgeR package of R was used to generate the expression matrix screen differential expression genes (DEGs) between the Sup-DLBCL group and the Sub-DLBCL group with the threshold of false discovery rate (FDR) set at 0.05, and the |log_2_-fold change| (|log_2_FC|) minimum threshold set at 2; the gene expression values were averaged when measuring the same gene in multiple probes. The differences between the two groups were statistically assessed by Student’s t-test; the *P*-values were adjusted for multiple-testing by the Benjamini–Hochberg (BH) method.

### 2.7. Functional and Pathway Enrichment Analysis

Functional enrichment analysis molecular function (MF), biological process (BP), and cellular component (CC) terms was conducted using the clusterProfiler package of R statistical software. KEGG (Kyoto Encyclopedia of Genes and Genomes) pathway enrichment analysis was also performed to identify the key biological pathways using the pathview package of R. Enrichment analyses were separately performed in the upregulated and downregulated genes. The *P*-value was set at <0.05 for inferring statistically significant enrichment of gene ontology (GO) or pathway terms, and the fold enrichment score was used to quantify the enrichment.

### 2.8. Gene Set Enrichment Analysis

The R package “clusterprofiler” was utilized to conduct gene set enrichment analysis (GSEA) to determine the statistically significant gene sets enriched in the different subgroups examined. The expression matrix of the TCGA dataset was analyzed using the annotation file “hallmark gene sets” in the Molecular Signatures Database (MSigDB). The cutoff values were set to *P* < 0.05, adjusted *P*-value < 0.05 and FDR < 0.25.

## 3. Results

### 3.1. Clinical Characteristics of Patients

A total of 9738 patients with early-stage nodal DLBCL had been reported to the SEER database from 2000 until 2014. The baseline information of the patients is summarized in Table 1. Most of the patients were white and had received chemotherapy but no surgery or radiotherapy. Statistically significant differences were observed for age, race, and Ann Arbor’s stage between the Sup-DLBCL and Sub-DLBCL groups, respectively. This study compared 6083 (62.5%) Sup-DLBCL patients and 3655 (37.5%) Sub-DLBCL patients, among which 5371 cases (55.2%) were males and 4367 cases (44.8%) were females. The average age of all, Sup-DLBCL, and Sub-DLBCL patients was 59.1, 56.7, and 63.2 years old, respectively. The incidence rate of Sup-DLBCL was higher than the one of Sub-DLBCL. Sub-DLBCL patients were older and had more patients over 60 years old compared to Sup-DLBCL patients. The median survival time for all, Sup-DLBCL, and Sub-DLBCL patients was 66.7 (range: 1.0–203.0), 72.6 (range:1.0–203.0), and 57.7 (range:1.0–203.0) months, respectively.

### 3.2. Survival Analyses

**Figure 2:**
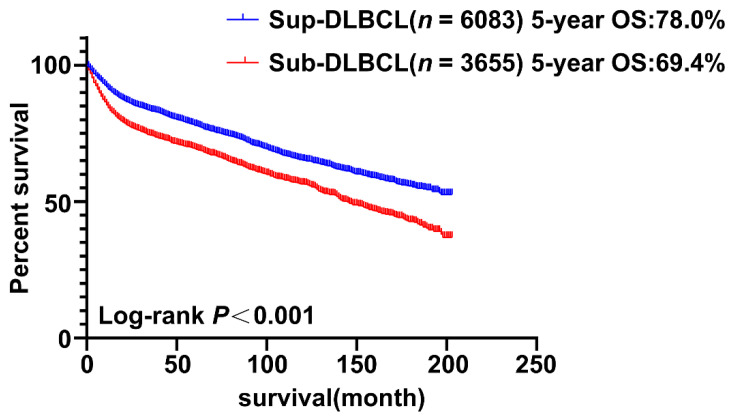
Kaplan–Meier curves of overall survival for patients with diffuse large B-cell lymphoma Sup-DLBCL (*n* = 8694) and Sub-DLBCL (*n* = 7158).

The Kaplan–Meier method was used to construct survival curve and the log-rank test was used to evaluate the differences among groups in order to explore the overall survival of patients with early-stage nodal DLBCL located on both sides of the diaphragm during the rituximab era. Table 2 presents the 5-year overall survival rate of patients with nodal DLBCL according to primary sites. Our results demonstrated that the 5-year overall survival rate was 74.8%. The 5-year OS rates of Sup-DLBCL and Sub-DLBCL were 78.0% and 69.4%, respectively (*P* < 0.001). The median survival time of the whole dataset was 185.0 months, while the Sup-DLBCL and Sub-DLBCL groups were undefined and 145.0 months, respectively. The overall survival of patients whose primary site was located under the diaphragm was worse than the one of the patients whose primary site was located on the diaphragm, as shown in Figure 2 and Table 2.

### 3.3. Propensity Score Matching

**Figure 3:**
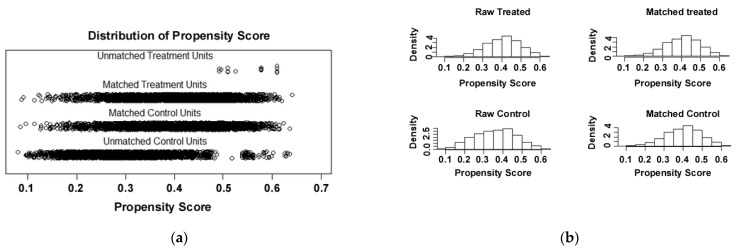
(**a**) Distribution map of the propensity score before and after matching. (**b**) Histogram of the propensity score before and after matching. Treated: Sub-DLBCL; Control: Sup-DLBCL.

The propensity score matching (PSM) of the baseline factors of the dataset examined was carried out to reduce the selection deviation. There were 3638 matches between the two groups. Figure 3a,b depicts the distribution map and histogram of the propensity score before and after matching. After PSM, all baseline factors were matched except the Ann Arbor’s stage, as shown in Table 3. Then, the matched data were analyzed with the Kaplan–Meier survival analysis and similar results were obtained. The 5-year overall survival rate and the median survival time of the whole matched data were 71.6% and 153.0 months, respectively. The 5-year OS rates of Sup-DLBCL and Sub-DLBCL were 73.5% and 69.6%, respectively (*P* < 0.001). The median survival time of Sup-DLBCL and Sub-DLBCL was 158.0 and 146.0 months, respectively, as shown in Table 2.

### 3.4. Adjusted Model

Multivariable Cox proportional hazard regression analysis was then performed to adjust for baseline factors that may impact the patient’s disease prognosis. The unadjusted HR of patients with the primary site located under the diaphragm was 1.48 (*P* < 0.001, 95%CI: 1.38–1.58) before PSM, while the unadjusted HR was 1.15 (*P* < 0.001, 95%CI: 1.07–1.24) after PSM. Only the meaningful factors from the univariate analysis were considered in the multivariate analysis. Thus, age, stage, race, and primary site were included in the multivariate analysis. Table 4 lists the univariate analysis before and after PSM, and Table 5 lists the unadjusted and adjusted models. Multivariate Cox proportional hazard regression analysis showed that before PSM, the overall survival of patients with primary site located under the diaphragm was worse than the one of the patients whose primary site was located on the diaphragm (*P* < 0.001, HR = 1.24, 95%CI: 1.16–1.33) after adjusting for age, stage, and race. After PSM, the overall survival of the Sub-DLBCL patients was still worse than the one of the Sup-DLBCL patients (*P* < 0.001, HR = 1.15, 95%CI: 1.07–1.24).

### 3.5. Identification of DEGs

**Figure 4:**
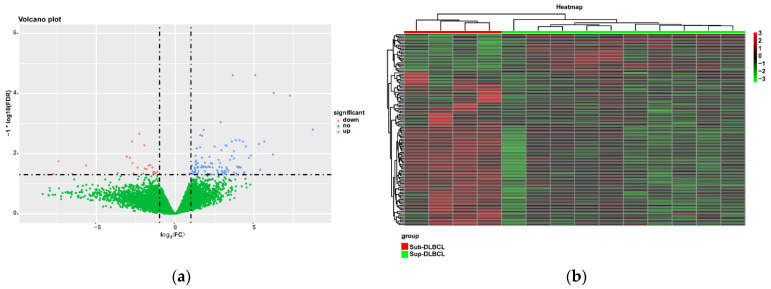
Differentially expressed genes (DEGs) between the supradiaphragm group and subdiaphragm group: (**a**) Volcano plot of DEGs; (**b**) heat map of DEGs. Differential-expressed genes are represented in rows, and samples are represented in columns. The expression value for each row was normalized by the z-score. Red stripes indicate high expression and green stripes indicate low expression of genes. Green bar represents Sup-DLBCL samples, while red represents Sub-DLBCL samples.

Differential expression genes (DEGs) between Sup-DLBCL group and Sub-DLBCL group were then identified using data from the TCGA database. A total of 130 DEGs were identified using the “edgeR” package with the following thresholds: FDR < 0.05 and |log_2_FC| > 1. 104 (80%) upregulated and 26 (20%) downregulated genes between the Sup-DLBCL group and the Sub-DLBCL group were found. The top 10 upregulated genes and 10 downregulated genes identified in the gene expression microarray study are presented in Table 6. In addition, volcano plots were generated to visualize the distribution of the DEGs and a heatmap with a dendrogram for clustering the DEGs was illustrated using the “ggplot2” package in R software (Figure 4a,b).

### 3.6. GO and KEGG Pathway Analysis for DEGs

**Figure 5:**
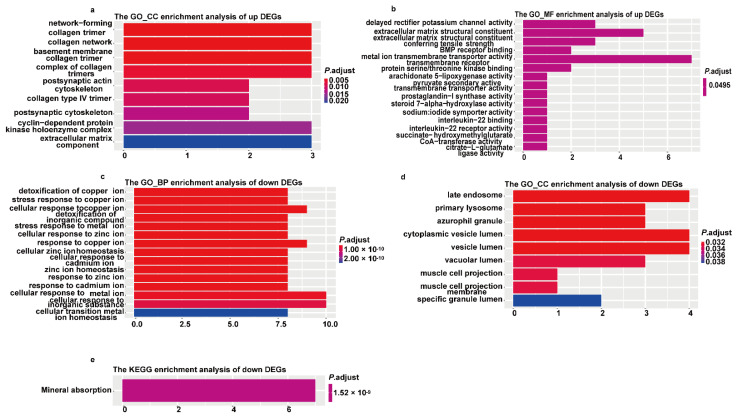
Gene ontologies (GO) analysis of DEGs between Sup-DLBCL and Sub-DLBCL: (**a**,**b**) cellular component (CC) and molecular function (MF) of gene ontology in upregulated genes; (**c**–**e**) cellular component (CC) and biological processes (BP) of gene ontology and KEGG for downregulated genes.

Functional enrichment analysis was applied to these DEGs to interrogate the biological functions of DEGs (Figure 5). Network-forming collagen trimer/collagen network/basement membrane collagen trimer were the most enriched GO terms in CC, and delayed rectifier potassium channel activity/extracellular matrix structural constituent/extracellular matrix structural constituent conferring tensile strength were the most enriched GO terms in MF for the upregulated genes, while no term was found enriched in BP and KEGG. Late endosome/primary lysosome/azurophil granule was significantly enriched in CC, detoxification of copper ion/stress response to copper ion/cellular response to copper ion was significantly enriched in BP, and only mineral absorption was significantly enriched in KEGG for the downregulated genes, while the remaining terms were insignificant in MF.

### 3.7. GSEA Analysis of the Gene Expression Files in the Sub-DLBCL Group versus the Sup-DLBCL Group

**Figure 6:**
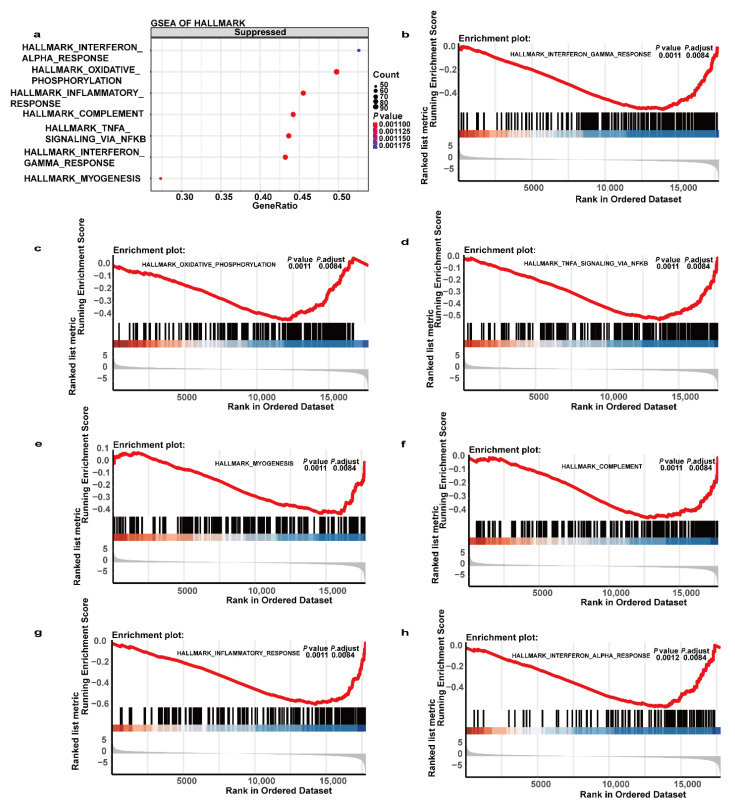
Gene sets enrichment analysis (GSEA) result of Sup-DLBCL patients compared to Sub-DLBCL patients. (**a**) GSEA analysis using hallmark gene sets was carried out. “Suppressed” indicates the pathways enriched in the Sup-DLBCL group. GSEA analysis showed that Sub-DLBCL was negatively associated with (**b**) interferon gamma response, (**c**) oxidative phosphorylation, (**d**) TNF-alpha signaling via NF-κB, (**e**) myogenesis, (**f**) complement, (**g**) inflammatory response and (**h**) interferon-alpha response.

The GSEA analysis was performed with the expression profile using a predefined “hallmark signature”. Seven gene sets were significantly enriched in the Sup-DLBCL group compared to the Sub-DLBCL group, with the top three including interferon gamma response (*P*.adjust = 0.008, NES = −2.005), interferon alpha response (*P*.adjust = 0.008, NES = −2.015), and inflammatory response (*P*.adjust = 0.008, NES = −2.230) (Figure 6).

## 4. Discussion

In the present study, clinical outcomes of early-stage nodal DLBCL were analyzed to investigate the prognostic impact of tumor location by classifying lymphoma into two groups: Sup-DLBCL and Sub-DLBCL. Our findings suggested that Sub-DLBCL was associated with worse 5-year OS compared to Sup-DLBCL (HR = 1.24; *P* < 0.001). The incidence of DLBCL under the diaphragm was lower, the age was higher, and there were more patients older than 60 years old in comparison with the Sup DLBL group. The OS of all patients in this study was 74.8%, in contrast to the previous studies which reported values in between 62% and 65.5% [19,21]. This difference could be attributed differences in the population’s inclusion criteria. Our results suggested that the prognosis of DLBCL patients in the Sub-DLBCL group was poorer.

PSM was also performed to reduce the selection deviation in order to obtain more reliable results. All baseline factors were matched after PSM while similar results were obtained showing that the prognosis of DLBCL patients in the Sub-DLBCL group was poorer than the Sup-DLBCL group (HR = 1.15; *P* < 0.001). The HR became smaller but more stable after adjusting for the influence of baseline factors, indicating that the baseline factors affected the results to a certain extent, but there were still significant statistical differences in the results, demonstrating that our results are robust.

Recent advances in genomic analysis allow formation of a comprehensive insight into the molecular heterogeneity of DLBCL and in stratifying risk for DLBCL. Currently, there are multiple classification systems available, including the international prognostic index (IPI), the cell-of-origin (COO) classification, and the capture double-hit lymphoma (DHL) and related subtypes [8,11,22,23,24]. Other attempts are under way in addition to the aforementioned categorization systems. For instance, based on the genomic aberrations, DLBCLs were divided into four subtypes or five subtype as mentioned in the background. In the present study, gene expression profile analysis using TCGA public database was conducted, including 10 Sup-DLBCL and 4 Sub-DLBCL samples, in an attempt to clarify the molecular changes between the two groups and to provide the molecular foundation for grouping the patients with early-stage nodal DLBCL. Finally, we identified 130 DEGs, with 104 upregulated and 26 downregulated. Among them, the tumor necrosis factor receptor superfamily, member 14 (TNFRSF14) is downregulated in the Sub-DLBCL, which may map this group to the previously described EZB subtype [8,10,25]. As mentioned in the introduction, the authors performed whole-exome sequencing and found that EZB subtype mainly harbored EZH2 mutations and BCL2 translocations, mapping to the previously described C3 and BCL2 clusters, and exhibits frequent inactivation of TNFRSF14, with inferior outcome to other GCB patients within GCB DLBCL. In the present study, we found that TNFRSF14 is downregulated in Sub-DLBCL by analyzing RNA-sequencing data and screening DEGs, which may have a similar biological function to its inactivation of EZB subtypes, explaining the worst outcome of Sub-DLBCL. Considering that COO and MYC/BCL2 status may not influence the outcome among patients with early-stage DLBCL treated with R-CHOP regimen [26,27,28,29,30,31], other factors (internal or external microenvironmental factors) may drive the poor prognosis of early-stage nodal DLBCL located under the diaphragm. This hypothesis, however, needs to be further confirmed in the future, as we only included early-stage nodal DLBCL patients while only RNA-sequencing data were analyzed and not DNA sequencing.

The DEGs generated by the comparison of the Sup-DLBCL and Sub-DLBCL groups were screened for further GO/KEGG analysis. The extracellular matrix-related terms were the most significantly enriched BP/CC/MF terms in upregulated genes. These genes have been reported to promote growth, survival, and invasion of cancer and to interact with fibroblast and immune cells to promote metastasis and impair treatment [32,33,34,35,36,37]. Most of the previous studies concerning the tumor microenvironment in DLBCL have focused on tumor-associated immune cells such as T cells, natural killer (NK) cells, and tumor-associated macrophages (TAMs) [38,39]. Few studies have given emphasis on extracellular matrix, an important part of tumor microenvironment. Recently, a group identified a genetic signature based on TME-related genes, comprising TIMP2, QKI, LCP2, LAMP2, ITGAM, CSF3R, and AAK1 [40], by calculating the abundance of immune–stromal components, and from this group they further obtained differentially expressed genes. In our research, we found that extracellular matrix structural constituents such as the network-forming collagen trimer and collagen type IV trimer were upregulated in the Sub-DLBCL group, which may contribute to the development of DLBCL located under diaphragm. In fact, studies have shown the effect of these extracellular matrix structural constituents on tumor progression. A study demonstrated that collagen IV can mediate LOXL2 to stimulate angiogenesis [41]. The type I-trimer collagen was found to be expressed in ductal infiltrating carcinomas and promote migration of tumor cells [42]. Our results suggested that the overexpression of extracellular matrix structural constituent may have contributed to the development of DLBCL located under diaphragm. More focus in the future should be given on the role of the extracellular matrix on early-stage nodal DLBCL. However, no significant KEGG pathway term was identified as significantly enriched in the upregulated genes. GO/KEGG enrichment analyses indicated that the downregulated DEGs were mainly manifested in ion balance in vivo, such as zinc and cadmium ions. Maintenance of ion homeostasis is primarily mediated by ion channels, which has been reported to be a tumor suppressor or an oncogene associated with tumor development [43,44]. Moreover, in our study, genes related to zinc, cadmium, and other ion homeostasis were downregulated in the Sub-DLBCL group with poor prognosis, which suggests that the ion homeostasis may be involved in early-stage nodal DLBCL as a tumor suppressor. At present, its role in DLBCL still remains unclear, and this is the part we should put more focus on in the future.

Besides the analysis of DEGs of Sup-DLBCL group versus Sub-DLBCL group set, this study also provides insightful GSEA results. Seven gene sets were enriched in the Sup-DLBCL group and not in the Sub-DLBCL group, including interferon gamma response, interferon alpha response, inflammatory response, myogenesis, oxidative phosphorylation, and complement and TNFα_signaling via NFκB. Interferons (IFNs) are cytokines that have antiviral, antitumor, and immunomodulatory properties, mediating immune response. IFN-α is a member of the type I IFN family, while IFN-γ is the lone member of the type II IFN family. IFN-α and IFN-γ are considered to have proapoptotic, anti-proliferative, and immune-related functions, such as promote myeloid cell activation and antigen presentation, which catalyzes the tumor’s elimination [45,46,47,48]. Sistigu et al. suggested that neoplastic cells can engage in a type I IFN response early after exposure to anthracyclines, and the therapeutic activity of anthracyclines relies on type I IFN signaling in neoplastic cells [49]. Although IFN-γ induces a broad spectrum of tumor-protective mechanisms, reason study has proved that IFN-γ-dependent senescence induction is a key mechanism required to protect against those cancer cells that escape from cytotoxicity [50]. CC-122, a new chemical entity termed as pleiotropic pathway modifier, has been reported to have anti-tumor effect in both the activated B-cell (ABC) and the germinal center B-cell DLBCL, mainly by inducing the degradation or short hairpin RNA–mediated knockdown of Aiolos and Ikaros. Moreover, it has been found to correlate with the increased transcription of interferon (IFN)-stimulated genes independent of IFN-α, -β, and -γ production and/or secretion [51]. Our results demonstrated that the Sup-DLBCL seems to have a more active interferon response presenting a better response to R-CHOP-based immunochemotherapy and a better prognosis than the patients with Sub-DLBCL. This result may imply an important method to stratify early-stage nodal DLBCL patients based on the level of interferon and a potential therapeutic target suggesting the potential future inclusion of IFN-γ or IFN-γ analogue in combined therapies for early-stage nodal DLBCL patients with tumors under the diaphragm. Further experiments and more effort are required to validate the therapeutic role of IFN-γ or IFN-γ analogue and to unravel the specific differentiated molecular biological mechanism between groups.

To the best of our knowledge, this is the first study providing a comprehensive descriptive analysis of patients with early-stage DLBCL based on the definition of lymph node distribution in Ann Arbor staging. The results of the present manuscript demonstrated that the Sub-DLBCL group presents worse prognosis, which confirms that when dealing with early-stage nodal DLBCL with tumors located under the diaphragm, we should be more vigilant, monitor more closely recurrence after treatment, and try to achieve cure or long-term survival. Currently, many studies have compared the clinical characteristics and survival differences between nodal and extranodal sites and between patients with different extranodal sites [21,52,53], but the comparisons of patients with different nodal sites remain limited. In the present manuscript, we thus mainly compare the survival differences of patients with different nodal sites and explore its underlying molecular mechanism. At present, it is known that the prognoses of the Sup-DLBCL and the Sub-DLBCL are different, perhaps partly because the diagnosing of nodal DLBCL under the diaphragm is more difficult and the symptoms are not specific, making the diagnosis more difficult. Our study provided additional evidence for these differences between Sup-DLBCL and Sub-DLBCL, attributing them to molecular mechanisms such as extracellular matrix and the regulation of inflammatory response.

This study has specific limitations since it is based on publicly available data: (1) Despite the use of propensity scores matching to control the selection bias for clinical observation studies, selection bias still exists. (2) We cannot determine the type of chemotherapy, the duration of chemotherapy, and the patient’s response status to treatment; the progression-free survival period cannot be obtained due to the limitation of the database, and thus we cannot conclude about the difference in response status of the two groups of patients to treatment. (3) There are no records of baseline performance status, B-symptoms, bulk disease, and lactate dehydrogenase levels in the SEER database, while limited information is available about treatments. Therefore, adjustments for these potential prognostic factors are not feasible.

## 5. Conclusions

Despite these limitations, our study has shown that the primary site of early-stage nodal diffuse large B-cell lymphoma affects its prognosis, and the prognosis of nodal DLBCL patients with tumors growing under the diaphragm is poorer. We screened DEGs by analyzing RNA-expression profiling data from the TCGA database and found several pathways, suggesting that the genetic characteristics between the different primary site of early-stage nodal diffuse large B-cell lymphoma is different. In clinical practice, the primary site of the nodal DLBCL growth should be considered comprehensively and the optimal treatment should be selected with reference to the primary site.

## Figures and Tables

**Table 1:** Demographic characteristics of patients with early-stage nodal diffuse large B-cell lymphoma.

Characteristics		Total	Anatomical Sites		*P*-Value
			Sup-DLBCL	Sub-DLBCL	
Patient, no. (%)		9738	6083 (62.5)	3655 (37.5)	
Gender, *n.* (%)					0.321
	Male	5371 (55.2)	3331 (54.8)	2040 (55.8)	
	Female	4367 (44.8)	2752 (45.2)	1615 (44.2)	
Age (mean)		59.1 (±17.6)	56.7 (±18.8)	63.2 (±14.7)	<0.001
age, *n*. (%)					<0.001
	≤60	4731 (48.6)	3267 (53.7)	1464 (40.1)	
	>60	5007 (51.4)	2816 (46.3)	2191 (59.9)	
Race, *n*. (%)					<0.001
	White	8234 (84.6)	5004 (82.3)	3230 (88.4)	
	Black	677 (7.0)	459 (7.5)	218 (6.0)	
	Other	827 (8.5)	620 (10.2)	207 (5.7)	
Stage, *n*. (%)					<0.001
	I	5880 (60.4)	3857 (63.4)	2023 (55.3)	
	II	3858 (39.6)	2226 (36.6)	1632 (44.7)	
Survival times (median, range)		66.7 (1.0–203.0)	72.6 (1.0–203.0)	57.7 (1.0–203.0)	<0.001
dead, *n*. (%)		3288 (33.8)	1843 (18.9)	1445 (14.8)	

Sup-DLBCL: supradiaphragm group; Sub-DLBCL: subdiaphragm group; *n*.: number.

**Table 2:** Overall survival (OS) of patients with early-stage nodal DLBCL according to primary sites.

Anatomical Sites	*n* (%)	Median Survival (Month)	5-Year OS (%)	*P*-Value
All	9738	185.0	74.8	<0.001
nodal of Sup-DLBCL	6083 (62.5)	undefined	78.0	
nodal of Sub-DLBCL	3655 (37.5)	145.0	69.4	
All (after PSM)	7276	153.0	71.6	<0.001
Sup-DLBCL (after PSM)	3638 (50.0)	158.0	73.5	
Sub-DLBCL (after PSM)	3638 (50.0)	146.0	69.6	

Sup-DLBCL: supradiaphragm group; Sub-DLBCL: subdiaphragm group; *n*: number; PSM: propensity score matching; OS: overall survival.

**Table 3:** Demographic characteristics of patients after PSM.

Characteristics		Total	Anatomical Sites		*P*-Value
			Sup-DLBCL	Sub-DLBCL	
Patient, *n*. (%)		7276	3638 (50.0)	3638 (50.0)	
Gender, *n*. (%)					0.906
	Male	4052 (55.7)	2029 (55.8)	2023 (55.6)	
	Female	3224 (44.3)	1609 (44.2)	1615 (44.4)	
Age (mean ± SD)		(±16.4)	63.3 (±15.79)	63.1 (±14.7)	0.689
age, *n*. (%)					0.649
	≤60	2908 (40.0)	1444 (39.7)	1464 (40.2)	
	>60	4368 (60.0)	2194 (60.3)	2174 (59.8)	
Race, *n*. (%)					0.422
	White	6418 (88.2)	3205 (88.1)	3213 (88.3)	
	Black	459 (6.3)	241 (6.6)	218 (6.0)	
	Other	399 (5.5)	192 (5.3)	207 (5.7)	
Stage, *n*. (%)					0.015
	I	4138 (56.9)	2121 (58.3)	2017 (55.4)	
	II	3138 (43.1)	1517 (41.7)	1621 (44.6)	
Survival times (median, range)		62.0 (1.0–203.0)	65.0 (1.0–203.0)	58.0 (1.0–203.0)	<0.001
dead, *n*. (%)		2764 (38.0)	1332 (18.3)	1432 (19.7)	

Sup-DLBCL: supradiaphragm group; Sub-DLBCL: subdiaphragm group; *n*: number.

**Table 4:** The univariate analysis before and after PSM.

Variable		Univariate Analysis before PSM		Univariate Analysis after PSM	
		HR (95%CI)	*P*-Value	HR (95%CI)	*P*-Value
Gender	Female	0.99 (0.92–1.06)	0.715	1.01 (0.93–1.09)	0.841
	Male	Reference		Reference	
Age	≤60	Reference		Reference	
	>60	3.72 (3.43–4.02)	<0.001	3.43 (3.12–3.78)	<0.001
Race	White	Reference		Reference	
	Black	1.15 (1.01–1.31)	0.034	1.15 (0.99–1.34)	0.061
	Other	0.83 (0.72–0.95)	0.007	1.08 (0.91–1.28)	0.358
Stage	I	Reference		Reference	
	II	1.21 (1.13–1.30)	<0.001	1.13 (1.05–1.22)	<0.001
Site	Sup-DLBCL	Reference		Reference	
	Sub-DLBCL	1.48 (1.38–1.58)	<0.001	1.15 (1.07–1.24)	<0.001

Sup-DLBCL: supradiaphragm group; Sub-DLBCL: subdiaphragm group; PSM: propensity score matching; HR: hazard ratio; CI: confidence intervals.

**Table 5:** Cox proportional hazards model for overall survival in patients with early-stage nodal diffuse large B-cell lymphoma: Only the meaningful factors in the univariate analysis are included in the multivariate analysis.

Overall Survival	HR (Sup-DLBCL vs. Sub-DLBCL)	95%CI		*P*-Value
		Lower	Upper	
Unadjusted	1.48	1.38	1.58	<0.001
Multivariable Cox adjusted	1.24	1.16	1.33	<0.001
PSM not-adjusted	1.15	1.07	1.24	<0.001
PSM adjusted	1.15	1.07	1.24	<0.001

Sup-DLBCL: supradiaphragm group; Sub-DLBCL: subdiaphragm group; PSM: propensity score matching; HR: hazard ratio; CI: confidence intervals.

**Table 6:** The top 10 upregulated genes and 10 downregulated genes identified in the gene expression microarray of 10 supradiaphragm patients and 4 subdiaphragm patients.

Caption	Gene	logFC	*P*-Value	FDR
upregulate				
	IGKV1D-16	8.727128	4.60 × 10^−7^	0.001584
	IGKV1-8	7.285481	2.27 × 10^−8^	0.000117
	MTCO3P12	6.25246	1.38 × 10^−8^	9.53 × 10^−5^
	CCNA1	6.19943	1.45 × 10^−5^	0.010673
	IGKV3-20	5.649626	2.71 × 10^−6^	0.003948
	UMODL1	5.645779	2.95 × 10^−6^	0.003948
	CLDN16	5.389993	0.000139	0.03425
	OR13A1	5.30567	4.15 × 10^−6^	0.004761
	SYBU	5.069182	1.96 × 10^−9^	2.44 × 10^−5^
downregulate				
	MT1H	−8.09573	0.000317	0.049648
	CPA6	−7.7316	0.000287	0.04742
	BARX1	−7.41026	3.62 × 10^−5^	0.017811
	RETN	−6.5198	0.000278	0.047004
	MT1M	−5.6572	6.33 × 10^−5^	0.024665
	NACA3P	−3.65231	0.000228	0.044334
	GNLY	−3.08913	1.90 × 10^−5^	0.012237
	MT1F	−3.07549	2.03 × 10^−5^	0.012692
	MT1E	−2.88335	2.41 × 10^−5^	0.013726
	MT1X	−2.74386	3.25 × 10^−6^	0.003948

logFC: log-fold change; FDR: false discovery rate.

## Data Availability

Publicly available datasets were analyzed in this study. These data can be found here: https://cancergenome.nih.gov/, accessed date: 20 March 2020; https://seer.cancer.gov/, accessed date: 20 March 2020.
